# Insights into the roles of macrophages in *Klebsiella pneumoniae* infections: a comprehensive review

**DOI:** 10.1186/s11658-025-00717-7

**Published:** 2025-03-26

**Authors:** Yangguang Li, Xuanheng Li, Wenqi Wu, Peizhao Liu, Juanhan Liu, Haiyang Jiang, Liting Deng, Chujun Ni, Xiuwen Wu, Yun Zhao, Jianan Ren

**Affiliations:** 1 Research Institute of General Surgery, Jinling Hospital, Affiliated Hospital of Medical School, Nanjing University, Nanjing, China; 2 Department of General Surgery, BenQ Medical Center, The Affiliated BenQ Hospital of Nanjing Medical University, Nanjing, 210009 China; 3 School of Medicine, Southeast University, Nanjing, 210000 China; 4 Clinical Translational Research Center for Surgical Infection and Immunity of Nanjing Medical University, Nanjing, China

**Keywords:** Macrophage, *Klebsiella pneumoniae*, PAMPs, PRRs, Cell death, Immunotherapy

## Abstract

*Klebsiella pneumoniae* (KP) infections represent a significant global health challenge, characterized by severe inflammatory sequelae and escalating antimicrobial resistance. This comprehensive review elucidates the complex interplay between macrophages and KP, encompassing pathogen recognition mechanisms, macrophage activation states, cellular death pathways, and emerging immunotherapeutic strategies. We critically analyze current literature on macrophage pattern recognition receptor engagement with KP-associated molecular patterns. The review examines the spectrum of macrophage responses to KP infection, including classical M1 polarization and the newly described M(Kp) phenotype, alongside metabolic reprogramming events such as glycolytic enhancement and immune responsive gene 1 (IRG1)–itaconate upregulation. We systematically evaluate macrophage fate decisions in response to KP, including autophagy, apoptosis, pyroptosis, and necroptosis. Furthermore, we provide a critical assessment of potential future therapeutic modalities. Given the limitations of current treatment paradigms, elucidating macrophage–KP interactions is imperative. Insights gained from this analysis may inform the development of novel immunomodulatory approaches to augment conventional antimicrobial therapies, potentially transforming the clinical management of KP infections.

**Graphical Abstract:**

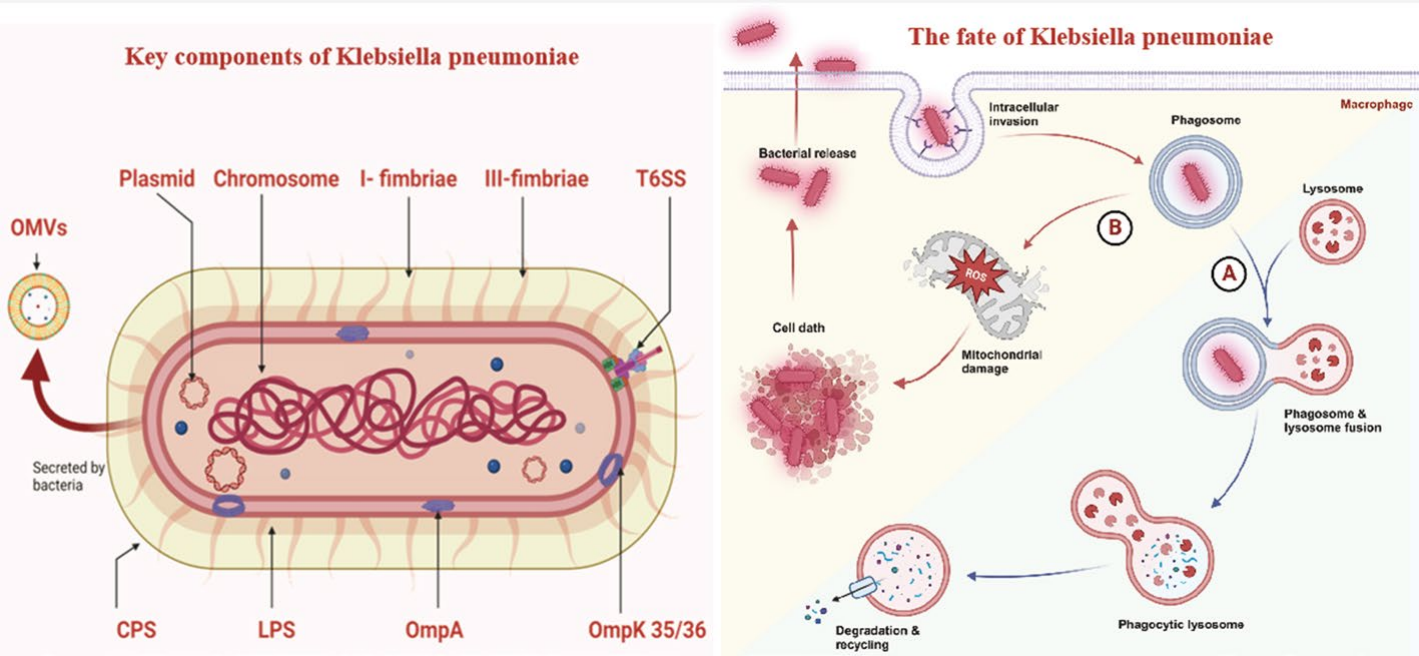

## Introduction

Macrophages are pivotal components of the innate immune system, serving as the first line of defense by surveiling, engulfing, and eliminating pathogens [1–4]. During microbial infections, macrophages recognize pathogen-associated molecular patterns (PAMPs) through pattern recognition receptors (PRRs), initiating intracellular signaling cascades and pathways [2, 5]. Concurrently, macrophages undergo processes such as autophagy, apoptosis, pyroptosis, and necroptosis to ensure immune-mediated killing of microbes and clearance of intracellular pathogens [6, 7]. However, bacterial infections can also trigger inflammatory storms through PAMPs, leading to excessive cell death and the release of large amounts of endogenous damage-associated molecular patterns (DAMPs) [5, 8]. Once an uncontrollable inflammatory storm occurs, even effective suppression of PAMP production and release through antibiotics or other infection control measures may not resolve the excessive inflammatory injury and multiple organ dysfunction caused by DAMPs [9, 10]. Therefore, it is crucial to consider the immune processes involved in *Klebsiella pneumoniae* (KP) infection during treatment.

First isolated in the late nineteenth century, KP is a facultative anaerobic pathogenic Gram-negative bacillus of *Klebsiella* spp. in Enterobacteriaceae [11, 12]. Currently, KP is gaining increasing attention worldwide, with the World Health Organization listing carbapenem-resistant KP (CRKP) as the highest priority critical pathogen in need of new therapeutic options since 2017 [12–14]. The SENTRY Antimicrobial Surveillance Program, spanning 20 years (1997–2016), identified KP as the third leading cause of bloodstream infections [15].

Recent evidence indicates that the interaction between KP and macrophages is highly complex, presenting challenges to conventional understanding [16–18]. KP can persist and replicate within macrophages, potentially polarizing them into a novel subtype termed M(Kp), with significant metabolic changes [16, 17, 19]. Summarizing the role of macrophages in KP infection is crucial to better understanding these dynamics. Given the rise of KP-induced severe inflammatory damage and the scarcity of effective antibiotic treatments, exploring macrophage–KP interactions could lead to the development of immunotherapies that synergize with antibiotics, ultimately improving patient outcomes [11, 20, 21].

This review provides a comprehensive overview of the key determinants of KP infection and the emerging immune processes involved. We discuss several stages of this intricate interaction, from macrophage recognition of KP via PRRs, macrophage polarization and metabolism, and innate immune responses (including autophagy, apoptosis, pyroptosis, and necroptosis), to identifying potential molecular targets for therapeutic intervention.

### Macrophage recognition of KP infection

**Fig. 1:**
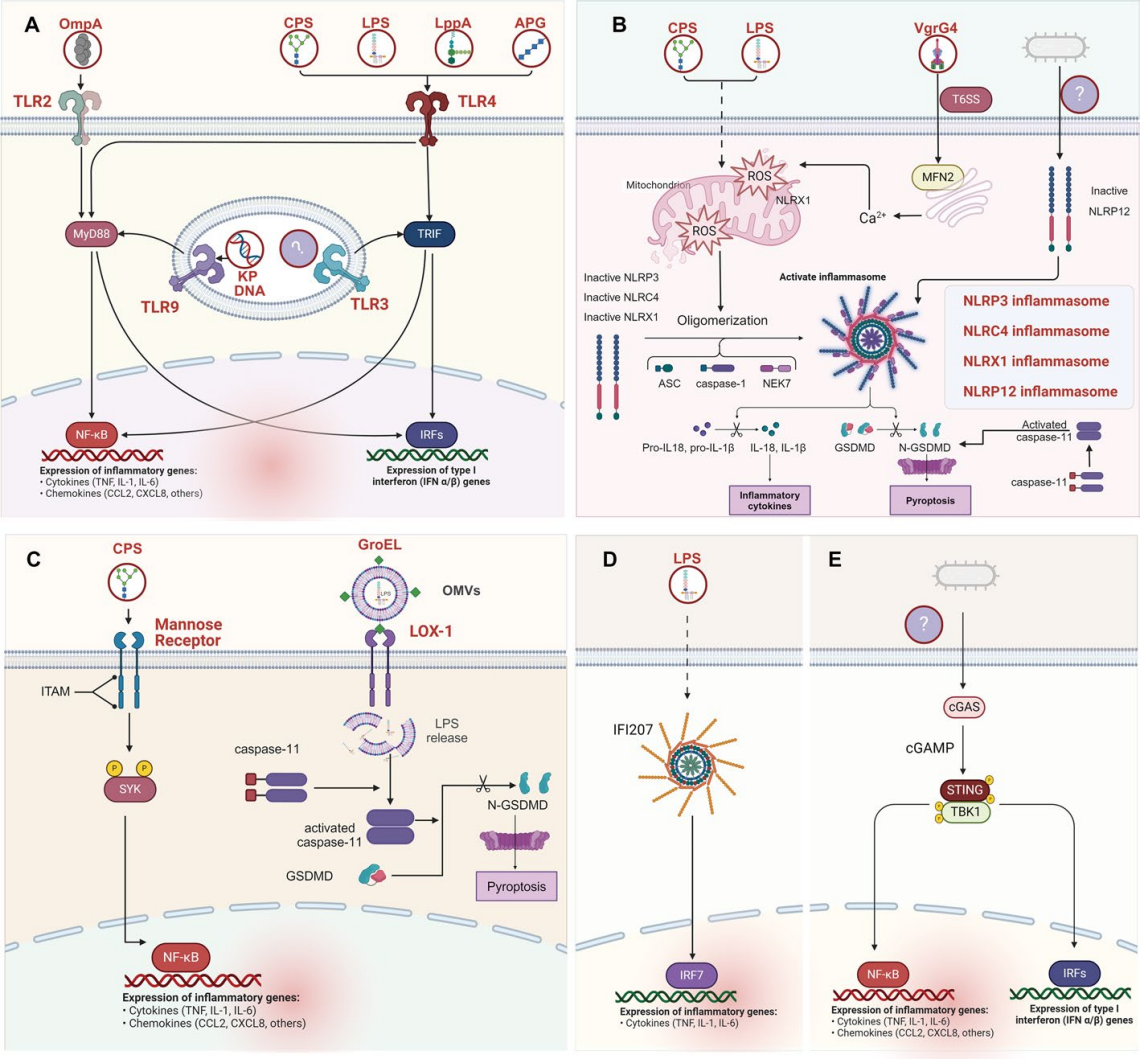
TLR, NLR, CLRs, ALRs, and cGAS pathways in *Klebsiella pneumoniae* infections. **A** Toll-like receptor (TLR) pathways; TLR4: recognizes the lipid A component of LPS, CPS, LppA (murein lipoprotein), and APG (acylpolygalactosyl), triggering immune responses via MyD88-dependent and TRIF pathways. TLR2: activated by the outer membrane protein OmpA. TLR3: recognizes unidentified components of KP. TLR9: activated by KP DNA, initiating immune responses. **B** Nod-like receptor (NLR) pathways; NLRP3 and NLRC4: KP triggers the NLRP3/NLRC4-caspase 1-IL-1β/HMGB1 pathway through LPS and CPS, dependent on mitochondrial dysfunction and increased ROS release. NLRP12: recognizes unidentified components of KP; NLRP12-deficient mice exhibit higher bacterial loads and decreased cytokine production. NLRX1: KP activates NLRX1 via the T6SS effector VgrG4, which binds to mitofusin 2, transferring Ca^2+^ from the ER to mitochondria, activating Drp1, and causing mitochondrial fragmentation. **C** C-type lectin receptors (CLRs); Certain KP CPS (K3, K7, K12) activate the macrophage mannose receptor (MMR). MMR activates the SYK pathway, promoting inflammation and antibacterial responses. LOX-1, a CLR within the dectin-1 cluster, responds to GroEL in KP OMVs under imipenem stress, leading to internalization of OMVs, release of free LPS, and activation of caspase-11-mediated pyroptosis. **D** AIM2-like receptor (ALR) pathways; IFI207, predominantly expressed in macrophages, plays a critical role in KP-induced lung infection by activating IRF7-mediated cytokine expression. Its activation is independent of dsDNA recognition but enhanced by LPS. **E** cGAS is a DNA sensor activating STING, leading to TBK1-mediated IRF3 and NF-κB pathway activation, inducing TNF, IL-1β,, and IL-6 expression. The specific KP component activating cGAS remains unclear, possibly derived from tissue damage DNA, warranting further investigation. Image created with BioRender.com, with permission

KP possesses distinct molecular and subcellular features not found in host cells, such as capsular polysaccharides (CPS), lipopolysaccharides (LPS), bacterial DNA, outer membrane proteins (OMPs), outer membrane vesicles (OMVs), and flagella [12, 14]. Macrophages detect and bind to PAMPs through PRRs, which primarily include Toll-like receptors (TLRs), nucleotide-binding oligomerization domain-like receptors (NLRs), retinoic acid-inducible gene I-like receptors (RLRs), C-type lectin receptors (CLRs), absent in melanoma 2 (AIM2)-like receptor (ALRs), and cyclic GMP–AMP synthase (cGAS) [2, 22]. This section summarizes the identified PRRs involved in KP infection and their corresponding KP PAMPs, outlining their roles (Fig. 1).

### TLR

To date, 10 Toll-like receptors have been identified in humans (TLR1-10), while 12 functional TLRs have been discovered in mice (TLR1–TLR9, TLR11–TLR13) [23, 24]. This diversity in TLRs reflects the evolutionary adaptation of the innate immune system to recognize a wide array of PAMPs. Accumulating evidence underscores the pivotal involvement of TLR4, TLR2, TLR3, and TLR9 in KP infections, and we delineate their functions in this context (Fig. 1) [17, 23, 25–35]. These specific TLRs play crucial roles in recognizing various components of KP and orchestrating the host immune response.

#### TLR4

TLR4, a member of the TLR family, recognizes the lipid A component of LPS from Gram-negative bacteria such as KP, triggering immune responses [23, 33]. Activation of TLR4 by KP initiates the MyD88-dependent pathway, ultimately promoting the activation of NF-κB and MAPK pathways, thereby facilitating the production of pro-inflammatory cytokines [27, 33]. Furthermore, KP can activate the TRIF pathway following TLR4 receptor activation, thereby stimulating downstream interferon regulatory factor 3 (IRF3) and IRF7, leading to the induction of type I interferon production [17, 34]. Research highlights the crucial role of TLR4 in macrophage-mediated defense against KP [25, 27, 30, 31, 33–36]. Mice lacking TLR4 exhibit suppressed inflammatory responses, elevated bacterial burden, and increased mortality following KP infection [30, 31, 33, 35].

Interestingly, besides LPS, the CPS of KP may also exert its effects by activating the TLR4 receptor, as observed in KN2 CPS and K1 CPS, while evidence for other CPS types remains unclear [27, 36]. When the CPS of KP strain KN2 is enzymatically cleaved by a serotype-specific bacteriophage enzyme, it produces a hexasaccharide repeating unit ({→ 3)-β-d-Glcp-(1 → 3)-[α-d-GlcpA-(1 → 4)-β-d-Glcp-(1 → 6)]-α-d-Galp-(1 → 6)-β-d-Galp-(1 → 3)-β-d-Galp-(1 →}) that demonstrates TLR4 activation capacity [36]. Experimental evidence demonstrates that CPS mediates the secretion of TNF-α and IL-6 by macrophages through the TLR4 pathway, a process dependent on the acetylation and *O*-acetylation of CPS [27]. Molecular docking simulations further confirm the potential binding between CPS and the TLR4 receptor [27, 37]. To date, the activation of the TLR4 receptor has been reported exclusively for KN2 and K1 CPS. Notably, KN2 CPS mediates this activation through a hexasaccharide repeating unit, whereas K1 CPS engages TLR4 through the Glc–Fuc–GlcA–Glc fragment [27, 36]. Purified CPS activates the EGFR via the MyD88-dependent pathway, along with the EGFR-dependent PI3K–AKT–PAK4–ERK–GSK3β signaling pathway. Additionally, K1-CPS induces the secretion of IL-1β mediated by the NLRP3 inflammasome via TLR4 [38]. In macrophages infected with K1-CPS-deficient mutants, LPS-deficient mutants, or K1-CPS and LPS double mutants, inflammasome activation is attenuated, as indicated by reduced IL-1β secretion levels [34]. However, IL-1β secretion in the double mutants does not decrease further compared with the single mutants and remains elevated relative to uninfected controls, suggesting the involvement of additional factors that warrant further investigation [34].

Murein lipoprotein (LppA) is one of the most important OMPs of KP [28]. Studies have found that NTUH-K2044 strains lacking LppA lose the ability to activate TLR4 receptors, indicating that LppA is directly or indirectly involved in the activation of TLR4 receptors [28]. Moreover, research has isolated a 34-kDa water-soluble acylpolygalactosyl (APG) from the membrane of KP through alkaline hydrolysis and delipidation [39]. Biotin-labeled APG, competitively binding with LPS, interacts with membrane receptors of monocytes, suggesting the potential of KP APG to activate TLR4, warranting further experimental investigation [39].

#### TLR2

TLR2 is considered the most promiscuous receptor among all TLRs [23, 40]. It can form heterodimers with other receptors such as TLR1 and TLR6, recognizing a wide range of ligands from various pathogenic sources [40]. The most extensively characterized ligands for TLR2 are lipoproteins found ubiquitously across all bacteria, with reports indicating that OmpA from KP can activate TLR2 receptors [28, 32]. Upon genetic deletion of TLR2 in a murine infection model with intranasal inoculation, bacterial loads were significantly elevated, particularly in the lungs and spleen, indicating the critical role of TLR2 in host defense against KP infection [30].

#### TLR3

TLR3, primarily responsible for recognizing dsRNA viruses, relies entirely on the adaptor molecule Toll/IL-1 receptor domain-containing adaptor inducing IFN-β to mediate the transcriptional induction of IFNs and chemokines, thereby establishing an antiviral host response. Unlike cytoplasmic RLRs, TLR3 is anchored to the endosomal membrane, where it detects the presence of dsRNA within the endosomal lumen. The minimum length requirement for dsRNA recognition by TLR3 is 40–50 base pairs. However, in addition to viral dsRNA, host or bacterial RNA can potentially activate TLR3, such as dsRNA released from damaged mitochondria triggering an immune response [41].

Interestingly, studies analyzing the role of TLR3 in KP infections have found that alveolar macrophages from TLR3−/− mice exhibit enhanced bactericidal and phagocytic abilities, resulting in significantly reduced mortality [26]. Furthermore, when TLR3 activation inhibitors or TLR3 neutralizing antibodies were employed, mouse survival rates improved [26]. These findings suggest a detrimental role of TLR3 in KP infection, although the precise mechanism by which TLR3 influences KP infection remains to be elucidated [26].

#### TLR9

TLR9 is a crucial intracellular DNA sensor predominantly located within endolysosomes, capable of being activated by bacterial-derived unmethylated cytosine–phosphate–guanine DNA or synthetic oligodeoxynucleotides containing unmethylated CpG motifs. This activation directly or indirectly initiates innate immune responses, aiding in the defense against pathogen invasion, including KP. Studies have demonstrated that mice lacking TLR9 exhibit significantly increased bacterial loads and higher mortality rates upon infection with KP [31]. The increased susceptibility of TLR9-deficient mice to KP infection suggests that TLR9 plays a critical role in recognizing KP DNA and initiating appropriate immune responses, with infection established via intratracheal administration [31]. Furthermore, the TLR9 antagonist oligodeoxynucleotide 4084-F can suppress the induction of TNF-α by KP (ST383), suggesting that plasmid DNA may activate the TLR9 signaling pathway [29]. These findings collectively emphasize the dual nature of TLR-mediated responses in KP infections.

### NLR

NLRs form a significant class of intracellular pattern recognition receptors in host cells [22, 42]. Research indicates that, upon phagocytosis of KP by macrophages, the pathogen and its components can reside within the cytosol, potentially activating NLRs [22, 42, 43]. NLRs typically consist of three domains: a central nucleotide-binding and oligomerization domain (NOD or NACHT), C-terminal leucine-rich repeat (LRR) sequences for ligand recognition, and N-terminal effector domains, such as caspase activation and recruitment domain (CARD) or pyrin domain (PYD) for protein interactions [22, 42]. Upon sensing and recognizing specific pathogen-associated molecular patterns, NLRs undergo self-oligomerization to form large signaling complexes, such as the inflammasomes assembled by NLRP3, NLRC4, and others [22, 42]. These complexes activate pathways including NF-κB, MAPK, and pyroptosis, leading to the release of pro-inflammatory cytokines such as TNF-α, IL-1β, and IL-18, which play critical roles in the inflammatory response. During KP infection, NLRP3, NLRC4, NLRP12, and NLRX1 play crucial roles in immune defense against KP infection [34, 44–48]. The activation of Nod-like receptor signaling is a characteristic feature of macrophages infected by KP, which will be discussed in detail in the following section (Fig. 1) [17, 34, 44–48].

#### NLRP3

NLRP3 can be activated by a wide range of stimuli, including mitochondrial dysfunction, and lysosomal damage [42, 43]. Studies have shown that KP can activate the NLRP3-caspase 1-IL-1β/HMGB1 pathway [34, 44]. Mice lacking NLPR3 exhibit reduced lung inflammation and lower survival rates compared with wild-type mice following KP infection [34]. The CPS and LPS of KP can mediate NLRP3 inflammasome activation through the induction of mitochondrial membrane permeability changes and mitochondrial ROS generation [34]. Research has also found that purple sweet potato anthocyanins (PSPAs) attenuate NLRP3 inflammasome activation by promoting mitophagy, which increases mitochondrial membrane potential and decreases mitochondrial ROS and mitochondrial DNA production, thereby reducing lung injury, limiting KP dissemination, and improving survival rates [47].

#### NLRC4

NLRC4, a key component of the innate immune system, belongs to the NLR family and plays a crucial role in the recognition of intracellular pathogens. NLRC4 can be activated by bacterial flagellin and the type III secretion system (T3SS) of *Salmonella*, initiating the NLRC4-caspase-1-IL-1β pathway [49, 50]. Interestingly, KP may activate the NLRC4 inflammasome through components distinct from flagellin or T3SS proteins, namely its capsular CPS and LPS [34]. NLRC4 plays a crucial role in host defense against KP infection. *NLRC4* gene knockout (KO) mice infected with KP via intratracheal administration exhibit downregulated IL-1β expression in the lungs, a response consistent with that observed in NLRC4 KO macrophages during KP infection, ultimately leading to reduced neutrophil chemoattractants and reduced survival rates [45]. The role of NLRC4 in KP infections opens up several avenues for further research and potential therapeutic interventions.

#### NLRP12

NLRP12 is a controversial member of the NLR family, inhibiting NF-κB pathways and forming inflammasomes to trigger IL-1β and IL-18 release [51]. A study conducted in 2017 demonstrated that NLRP12 inflammasomes, formed during high-dose KP ATCC 15380 (74,000 CFU) infection, do not affect the clearance or dissemination of KP in an acute lung infection model [52]. However, in NLRP12-deficient bone-marrow-derived macrophages (BMDMs), stimulation with purified KP LPS results in increased TNF-α and IL-6 levels compared with wild type (WT), without promoting IL-1β maturation or release [52]. Further research by Shanshan Cai et al. revealed that *NLRP12*−/− mice infected with 1000 or 10,000 CFU of ATCC 43816 via the intratracheal route exhibited higher CFU counts in the lungs, liver, and spleen [53]. Additionally, reduced NF-κB activation and decreased levels of cytokines (TNF-α, IL-6, and IL-1β) and the neutrophil chemoattractant CXCL2/MIP-2 were observed in the infected *NLRP12*−/− mice [53]. Transplanting WT BMDMs into *NLRP12*−/− mice restored survival rates, reduced bacterial loads in the lungs and spleen, and normalized neutrophil influx and cytokine/chemokine expression in the lungs [53]. Overall, the role of NLRP12 in KP infections remains inconsistent across studies. However, the findings by Shanshan Cai et al. underscore the critical importance of myeloid-derived NLRP12 in mounting an effective defense against KP infection [53].

#### NLRX1

NLRX1 is a unique member of the NOD-like receptor family, distinguished by its localization to mitochondria [54]. It was the first reported NLR to be associated with this critical cellular organelle, highlighting its potential importance in linking innate immunity with mitochondrial function. NLRX1 plays crucial regulatory roles in ROS generation, mitochondrial damage, autophagy, and apoptosis [54]. The type VI secretion system (T6SS) of *Klebsiella pneumoniae* is a versatile protein secretion apparatus that plays a critical role in interbacterial competition, host cell interactions, biofilm formation, antibiotic resistance, and virulence regulation. By delivering effectors into competing bacteria or host cells, it enhances the survival and pathogenicity of KP. Recent studies have revealed that KP can activate NLRX1 through its T6SS effector VgrG4 in eukaryotic cells, including yeast and the human lung carcinoma cell line A549, thereby restraining NF-κB inflammatory responses [48]. Macrophages, as eukaryotic cells, have not been specifically validated for this mechanism [48]. The specific mechanism involves the binding of VgrG4 to mitofusin 2, an endoplasmic reticulum protein, prompting the transfer of Ca^2+^ from the ER to mitochondria, activating the mitochondrial fission regulator Drp1, thereby causing fragmentation of the mitochondrial network [48].

### CLR

CLR constitute a class of innate immune receptors that are crucial for tailoring immune responses to pathogens [55, 56]. Two CLRs have been identified as associated with KP infection in previous studies: the macrophage mannose receptor (MMR) and lectin-like oxidized low-density lipoprotein receptor-1 (LOX-1) (Fig. 1) [55, 57, 58].

#### MMR

MMR, also known as CD206, recognizes and binds specific carbohydrate molecules through its extracellular domain [59, 60]. MMR plays a critical role in pathogen recognition, antigen presentation, and maintaining homeostasis, primarily inducing inflammation and antibacterial processes through the SYK kinase [61]. Recent research has shown that KP can activate SYK kinase in macrophages, including THP-1 cells, RAW264.7 cells, and BMDMs, potentially through the activation of the MMR [62]. Studies conducted in the late twentieth century revealed that certain capsular polysaccharides, such as those from KP serotypes K3, K7, and K12, can activate the MMR and subsequently the innate immune system [57]. However, the most common serotypes, K1 and K2, do not activate the MMR [57, 63].

#### LOX-1

LOX-1 is a receptor within the dectin-1 cluster, a subgroup of CLRs that also includes MICL, CLEC-2, CLEC-12B, CLEC-9A, MelLec, and Dectin-1 [55]. Interestingly, recent studies have found that, under imipenem stress, multidrug resistant (MDR)-KP exhibits a significant increase in GroEL protein within its secreted OMVs [58]. This increase activates LOX-1 receptors on the surface of macrophages, triggering pyroptosis and exacerbating the inflammatory response, characterized by elevated IL-18, IL-1β, TNF, and immune cell infiltration into the lungs [58]. Furthermore, both in vivo and in vitro experiments have shown that using OMV production inhibitors or suppressing LOX-1 expression significantly reduces the inflammatory response and decreases mouse mortality [58].

### ALR

ALRs are a novel type of PRRs that feature an N-terminal PYD and one or two C-terminal DNA-binding HIN domains, allowing them to sense intracellular dsDNA and activate inflammasomes or induce type I IFN production [22, 64]. Recently, Marcin Baran et al. identified IFI207, a receptor in the ALR family primarily expressed in macrophages, as playing a crucial role in the pathogenesis of KP infection (Fig. 1) [64]. Deletion of IFI207 significantly reduces the severity of KP-induced lung infection [64]. Mechanistically, IFI207 colocalizes with active RNA polymerase II and the nuclear transcription factor IRF7, subsequently activating IRF7-mediated cytokine expression, including TNF-α, IFN-β, and IL-6 [64]. Notably, its activation is independent of dsDNA recognition, and LPS can increase its expression [64]. Beyond mediating the production of inflammatory cytokines, IRF7 may also transcriptionally regulate host genes required for the phagocytosis of KP, reducing macrophage uptake of the bacterium [64]. In summary, KP infection activates IFI207, an ALR-type PRR in macrophages, leading to the production of inflammatory cytokines such as TNF-α [64].

### cGAS

cGAS is a cellular DNA sensor that primarily recognizes dsDNA to activate the innate immune response [65, 66]. Upon binding to DNA, cGAS catalyzes the production of cGAMP, which acts as a second messenger to activate the stimulator of interferon genes (STING) [65, 66]. The carboxyl terminus of STING recruits and activates TANK-binding kinase 1 (TBK1), which subsequently activates downstream IRF3-type I interferon and NF-κB pathways, inducing the expression of interferons and inflammatory cytokines such as TNF, IL-1β, and IL-6 (Fig. 1) [65–67].

KP infection can lead to the activation of the cGAS-STING signaling pathway. cGAS(−/−) and STING(−/−) mice exhibit reduced production of type I interferons, although there is no direct evidence that specific components of KP directly activate cGAS [68]. Interestingly, recent studies have revealed the role of STING in KP infection, showing that the absence of STING promotes the clearance of KP, as determined by intranasal infection of STING−/− mice, which, at 24 h post-infection, showed a 58% reduction in bacterial load in the lungs compared with wild-type infected mice [69]. This reflects the complex role of type I interferons in KP infection. Elevated type I interferon levels may induce the production of IL-10, which in turn promotes the survival of KP within macrophages [17, 69, 70].

## Modulation of macrophage phagocytosis by KP components

### CPS

The CPS of KP forms a dense polysaccharide fiber layer approximately 160 nm thick, primarily composed of linear or branched repeating units consisting of two to seven monosaccharides, providing effective protection against adverse environmental conditions [12, 14, 19]. Numerous studies have demonstrated that the CPS of KP serves as an effective barrier, preventing bacterial phagocytosis by macrophages and enhancing resistance to intracellular killing [18, 71–75]. The K1 and K2 serotypes represent the predominant highly virulent strains, constituting more than 80% of virulent isolates and commonly demonstrating elevated pathogenicity compared with other serotypes [11, 12, 76]. The increased virulence of K1 and K2 strains may be attributed to their enhanced resistance to phagocytosis and killing by macrophages [16, 72]. Recent studies have revealed that CPS promotes the survival of KP in the bloodstream by protecting bacteria from capture by liver-resident macrophages known as Kupffer cells [74]. Previous studies have found that the absence of mannose or sialic acid residues in the capsule structure of K1 and K2 serotypes allows evasion from recognition by mannose/lectin receptors, thereby impeding effective lectin-mediated phagocytosis and subsequent pro-inflammatory signaling [57, 77]. Additionally, the presence of sialic acid residues on the surface of K1 and K2 CPS may mimic host-derived sialic acid, allowing evasion of macrophage attack [57, 77, 78]. Furthermore, K2 serotype strains suppress macrophage phagocytosis through sialylation of the terminal ends of CPS [78].

Furthermore, it is interesting to note that certain strains of KP may use CPS to partially shield their LPS from detection by TLRs [79]. Studies have shown that strains with K1, K10, and K16 antigens can mask their LPS with CPS to prevent receptor binding, whereas other strains cannot, including those expressing K2 antigens [79]. This indicates that the role of CPS in helping KP evade macrophage-mediated innate immunity is varied and capsular serotype dependent, warranting further investigation.

### LPS

LPS, also known as endotoxin, is an essential component of the outer leaflet of the outer membrane in all Gram-negative bacteria [80]. It consists of a lipid A moiety, a core oligosaccharide unit, and a highly variable polysaccharide known as the O antigen, of which there are nine types, with O1 being the most common [14, 80]. Typically, LPS is a critical virulence factor for bacteria, with the lipid portion serving as a potent immune activator that triggers the TLR4 signaling pathway, leading to the production of cytokines and chemokines [14, 80]. However, KP can modify its LPS to evade detection by certain immune receptors, thereby circumventing innate immunity [81]. For instance, in a murine infection model with intranasal inoculation, KP can modify lipid A via 2-hydroxylation, dependent on the PhoPQ-regulated oxygenase LpxO, effectively suppressing innate immune responses and increasing its virulence in the lung [81]. It is important to note that mutations in the *O*-polysaccharide of LPS in KP do not affect phagocytosis by mouse alveolar macrophages [72].

### OmpA

The OmpA protein family, a group of heat-modifiable, surface-exposed porins, is one of the crucial OMPs in KP [32, 82–85]. Studies have demonstrated that deletion of OmpA in KP results in increased IL-8 production in bronchial epithelial cells, along with elevated levels of TNF-α and IL-6 in mouse lungs, suggesting a potential role for OmpA in suppressing cytokine secretion by macrophages [86]. Conversely, OmpA has been shown to activate TLR2 signaling in macrophages, thereby initiating the innate immune response [32]. Purified OmpA protein can effectively induce the upregulation of cytokine and chemokine production in murine lungs following intratracheal injection [83]. Hence, various forms of KP virulence factors may exhibit interconnected and intricate roles during infection.

### OmpK 35/36

OmpK35/36 are another outer membrane protein of KP, serving as crucial porins for antibiotic influx into the bacterium, and mutations in these proteins can lead to reduced antibiotic sensitivity in KP [72, 87–89]. However, such mutations have also been associated with a significant increase in mortality following KP infection, probably owing to their role in enhancing virulence by inhibiting phagocytosis by immune cells [72, 90]. Jiun-Han Chen et al. found that infection with OmpK36-deficient mutants resulted in increased phagocytosis of KP by immune cells as indicated by an increased number of fluorescently labeled bacteria engulfed, suggesting that the absence of OmpK36 alters the surface structure of the bacterium, potentially modifying its interaction with phagocyte receptors and promoting increased phagocytosis [90]. However, the precise mechanism underlying this process remains to be fully elucidated. Moreover, it is crucial to note that, while OmpK35/36 increases bacterial resistance to phagocytosis and virulence, it also facilitates antibiotic entry into the bacteria [84, 91]. Consequently, the absence of OmpK35/36 can lead to the emergence of multidrug-resistant strains, such as CRKP strains [84, 91].

### Macrophage polarization and metabolism

#### Macrophage polarization

##### M1 polarization

It is generally believed that, upon recognizing invading KP through PRRs, macrophages rapidly undergo classical activation, resulting in their polarization to the M1 phenotype, thereby establishing a robust immune response [1, 4, 5, 92]. A critical aspect of macrophage polarization is the activation of NF-κB, a key transcription factor orchestrating the inflammatory immune response to various stimuli [92, 93]. As previously mentioned, the LPS and CPS of KP engage TLR4, leading to the activation of NF-κB, which subsequently promotes the transcription of M1 marker genes [34, 92, 93]. Additionally, IRF3 contributes to M1 macrophage polarization by inducing the production of type I IFNs [1, 4, 5, 92, 93]. IFNs subsequently activate the transcription factor STAT1 via the interferon-alpha/beta receptor (IFNAR), driving the transcription of M1 marker genes such as CXCL9 and CXCL10 [1, 4, 5, 92, 93].

Studies indicated that, following KP infection, macrophages rapidly activate the NF-κB and interferon signaling pathways to promote M1 polarization [17, 34, 70]. This dual activation underscores the essential role of these pathways in orchestrating the early immune response to combat bacterial infection.

##### M(Kp) polarization

**Fig. 2:**
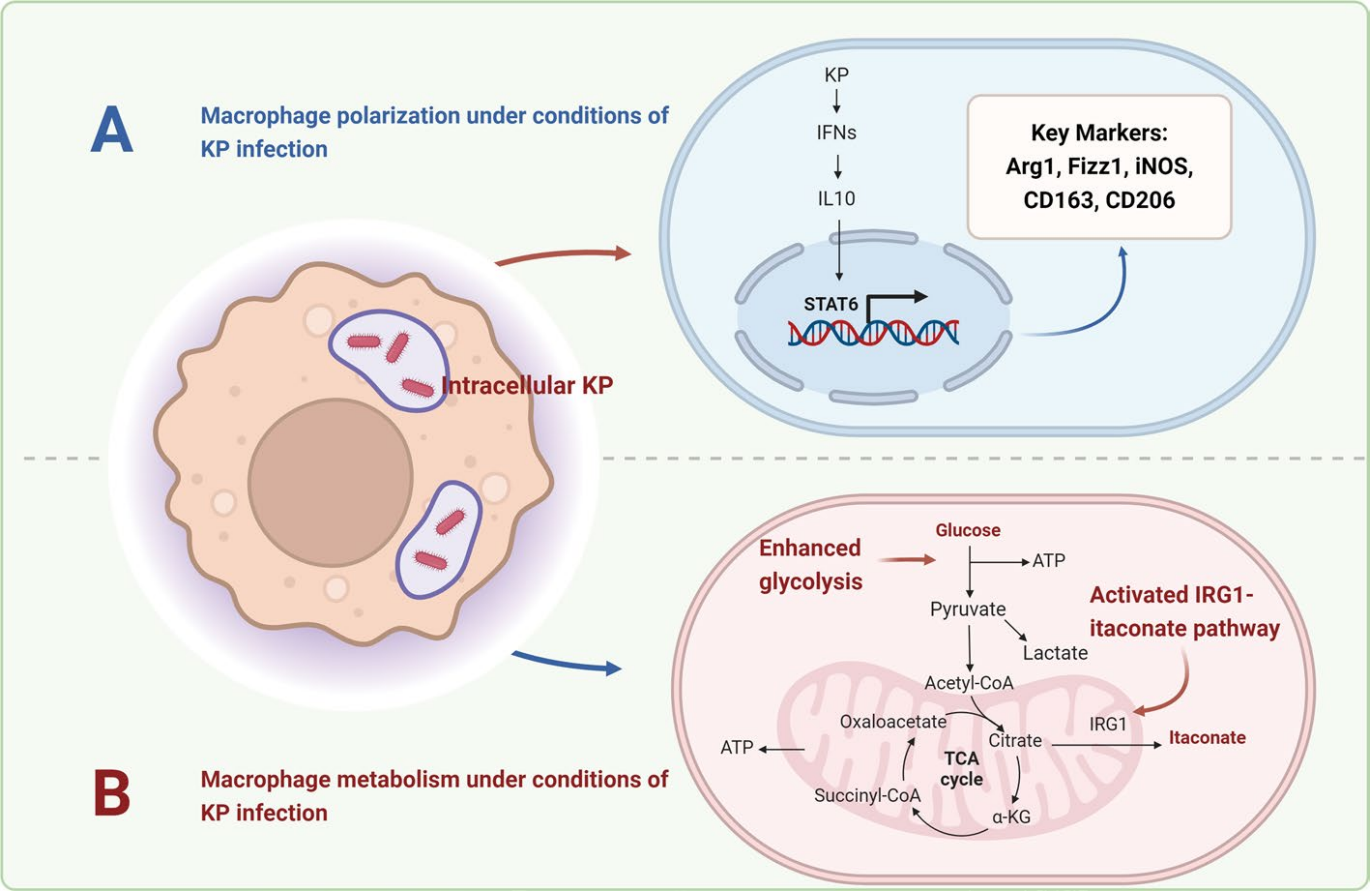
Macrophage polarization and metabolism in *Klebsiella pneumonia* infections. **A** M(Kp): a newly identified M2-like state following KP infection, characterized by upregulation of anti-inflammatory markers (Arg1, Fizz1, iNOS, CD163, and CD206) via STAT6 activation. **B** Metabolic reprogramming of macrophages infected with KP include enhanced glycolysis and the activation of the immune responsive gene 1 (IRG1)–itaconate anti-inflammatory pathway, a hallmark of tricarboxylic acid cycle (TCA) rearrangement. Image created with BioRender.com, with permission

Recent studies have identified a novel macrophage polarization state termed M(Kp) that emerges following infection with KP [17]. This state is characterized as an M2-like anti-inflammatory polarization, which is distinct from any known M2 subtype and is specifically driven by activation of the TLR2/4-type I IFN–IL-10–STAT6 signaling axis [17]. Key markers of M(Kp) polarization include Arg1, Fizz1, iNOS, CD163, CD206, and genes regulated by type I IFN and IL-10 signaling, along with a reduction of MHC-II and iNOS expression (Fig. 2) [17, 19]. Notably, the induction of the M(Kp) phenotype is specifically mediated by KP’s CPS, rather than its LPS, a mechanism that facilitates the bacterium’s intracellular persistence and survival within macrophages [17]. In contrast, STAT6 deficiency significantly enhances the clearance of KP in macrophages, underscoring the critical role of this signaling pathway in macrophage–KP interactions. Consistent with these findings, accumulating evidence indicates that KP can persist and replicate within macrophages, potentially driven by its CPS [16, 18, 94].

Beyond KP, *Salmonella typhimurium* has been shown to exploit TLR2/4-mediated signaling pathways to facilitate its intracellular survival within macrophages, while *Listeria monocytogenes* utilizes type I IFN signaling to establish persistence in host cells for survival [95, 96]. However, to date, no studies have reported that other bacterial species can induce a macrophage polarization state analogous to the unique M(Kp) phenotype. The newly discovered M(Kp) polarization state, whose phenotypes are complex and not strictly dichotomous unlike the widely used M1 (pro-inflammatory) and M2 (anti-inflammatory) classifications, warrants further in-depth investigation to fully elucidate its universality, underlying mechanisms, and implications in KP infection.

### Macrophage metabolism

When macrophages are subjected to infections, inflammation, or other external stimuli, they undergo metabolic reprogramming to meet their energy and biosynthetic demands. Amy Dumigan et al. reported that enhanced glycolysis is the primary metabolic pathway in M(KP) polarization, and inhibiting glycolysis increases the clearance of KP within macrophages, thereby enhancing their killing ability (Fig. 2) [17]. Pretreatment with 2-deoxy-d-glucose (2DG), a glycolytic inhibitor, significantly enhances the clearance of KP within macrophages, probably owing to increased autophagosome-lysosome fusion, as evidenced by elevated co-localization of KP-containing vacuoles with lysosomal markers [17]. Additionally, studies have found that KP ST258 can activate host glutaminolysis and fatty acid oxidation, creating an oxidant-rich microenvironment, which the bacteria adapt to by upregulating the type VI secretion system [97]. However, this microenvironment also leads to activation of the potent immune responsive gene 1 (IRG1)–itaconate anti-inflammatory pathway in immunometabolism, counteracting the inflammatory imbalance caused by KP infection [97]. Itaconate, which is derived from TCA cycle, has been shown to inhibit the expression of key protein kinases involved in various inflammatory responses, such as STING and Kelch-like ECH-associated protein 1, exerting anti-inflammatory and antioxidant stress effects [98–100]. Studies have demonstrated that the absence of STING, as observed in STING−/− mice following intranasal infection, enhances KP clearance from the lungs, with a significant reduction in bacterial load highlighting the potential of itaconate to promote bacterial clearance through STING inhibition, although the exact mechanism remains to be fully elucidated. Moreover, the accumulation of itaconate due to KP infection can alkylate and modify SYK, a key inflammatory kinase, regulating macrophage immunity and mitigating immune damage caused by KP infection [62]. Mice deficient in Irg1 show significantly increased bacterial loads and tissue damage in sepsis and pneumonia models [62, 97]. Overall, the metabolic characteristics of macrophages infected with KP include enhanced glycolysis and the activation of the IRG1–itaconate anti-inflammatory pathway, a hallmark of TCA cycle rearrangement.

### Macrophage innate defenses and regulation

#### Autophagy

Autophagy is considered a primary form of innate immunity against invading microorganisms, involving a collection of processes enabling cells to digest cytoplasmic components, including direct capture of intracellular microbes to exert bactericidal effects [7, 101]. During engulfment, LC3 is conjugated to the phagosome membrane, promoting autophagosome maturation into autolysosomes for pathogen degradation [7, 101, 102]. Throughout this process, key autophagy factors encoded by autophagy-related genes (ATGs) regulate the initiation, elongation, and maturation of autophagosomes at different stages [7, 101, 103]. Studies have revealed that KP can activate ATG7, associated with autophagosomal membrane elongation, triggering autophagy [104, 105].

As previously mentioned, various components of KP, including LPS, CPS, and APG, can activate the TLR4 receptor to exert their effects [17, 28, 30, 31, 33–36, 39]. The TBK1–NF-κB pathway induced by TLR4 is a key molecular driver of autophagy activation, directly inducing the expression of genes or proteins involved in the autophagic machinery, such as Beclin 1, the BAG3-HspB8 complex, ATG5, and LC3 [106]. Consistently, TLR4 has been shown to recognize LPS of KP and participate in autophagy activation during KP infection [104, 105]. NLRs are also essential for inducing autophagy, with Nod1 and Nod2 responding to bacterial peptidoglycans in the cytosol by recruiting ATG16L1 to the plasma membrane at the bacterial entry site, guiding autophagic mechanisms [107]. Beyond Nods, other NLRs interact with autophagy mechanisms; specifically, NLRX1 acts on the mitochondrial chaperone Tu translation elongation factor, interacting with Atg5–Atg12 and Atg16L1 to promote autophagy [46]. Considering that KP can activate NLRX1 through its T6SS effector VgrG4, thereby limiting the NF-κB inflammatory response, it is plausible that KP’s T6SS may also induce autophagy via NLRX1 [46, 48]. The cGAS–STING signaling pathway, in addition to inducing TBK-1 signaling and type I IFN production, can transduce signals from cytosolic DNA receptors to induce autophagy [108]. KP infection can activate the cGAS–STING signaling pathway, but whether KP induces autophagy via STING remains unclear [68, 69]. Recent studies have also suggested that KP may induce host protein misfolding via the PI3K–AKT–mTOR signaling pathway, thereby activating cellular autophagy and oxidative stress [36].

Interestingly, KP can proliferate stably within macrophages, potentially evading host cell autophagy [16, 18, 94]. Evidence suggests that engulfed KP fails to fuse with intracellular lysosomes, i.e., autophagolysosome formation is impaired following KP infection [16, 18, 94]. The specific mechanism may involve KP interfering with autophagy via the PI3K–AKT–Rab14 pathway, and autophagy blockade results in reduced clearance of KP within alveolar macrophages [18].

Autophagy plays a crucial role in macrophage resistance against KP infection [104, 105]. *ATG7* KO mice exhibit increased susceptibility to KP infection, reduced bacterial clearance, enhanced lung injury, and decreased survival rates [104, 105]. The mechanism involves ATG7 competitively inhibiting the interaction between p-IκBα and ubiquitin, resulting in reduced ubiquitination of p-IκBα, promoting NF-κB translocation to the nucleus, and exacerbating inflammatory damage [104, 105]. Additionally, when macrophage autophagy is inhibited by bafilomycin A1, intracellular growth of KP is enhanced [109, 110]. Similarly, in mice treated with the autophagy-enhancing mutant Becn1F121A or Beclin-1 activating peptide (Tat-beclin-1 peptide), the adverse outcomes of KP-induced sepsis are significantly alleviated [111]. Furthermore, 1,25-(OH)_2_-D_3_ can induce autophagy through the activation of ATG16L1-mediated autophagy, aiding macrophages in the clearance of KP [110].

#### Apoptosis

Apoptosis, a highly regulated form of programmed cell death, plays a crucial role in maintaining cellular homeostasis by eliminating unnecessary or defective cells [112, 113]. This process is orchestrated through two primary pathways: the intrinsic mitochondrial pathway and the extrinsic death receptor pathway. The intrinsic pathway is governed by Bcl-2 family proteins, which modulate mitochondrial outer membrane permeabilization, leading to the release of pro-apoptotic factors such as cytochrome *c*. Conversely, the extrinsic pathway is initiated by the binding of ligands to death receptors, resulting in the formation of the death-inducing signaling complex. Both pathways converge on the activation of caspase cascades, ultimately executing the apoptotic program.

The role of apoptosis in KP infections appears to be less significant, as reports indicate that KP primarily induces nonapoptotic cell death [18, 114]. This form of cell death is characterized by loss of membrane integrity, significant damage to phagolysosomes, and activation of caspase-1, with some cells releasing caspase-1-regulated extracellular traps. However, macrophages that phagocytose KP may undergo apoptosis due to the failure of phagosomes to fuse with lysosomes [18, 114]. Considering that KP T6SS effector protein VgrG4 colocalizes with MFN2, inducing the transfer of calcium from the endoplasmic reticulum to the mitochondria and activating Drp1, which leads to mitochondrial network fragmentation, it remains unclear whether the disruption of mitochondria and the subsequent release of cytosolic contents can trigger apoptosis [48]. Regarding the extrinsic signaling pathway, KP can activate macrophages to secrete TNF-α, which aids in clearing KP from the host lungs [115, 116]. However, it is unclear whether elevated TNF-α activates TNFR1 and caspase-8 to trigger apoptosis, thus increasing susceptibility to KP infection.

KP can inhibit apoptotic signaling pathways via factors such as its CPS, thereby resisting host bactericidal actions [114, 117]. While this effect has been mainly reported in neutrophils rather than macrophages, the capsule, as a significant virulence factor, can inhibit apoptosis by decreasing the Bax/Bcl-2 ratio and upregulating Mcl-1 expression [114]. This helps the bacteria evade destruction by phagocytes, allowing survival and dissemination within the host [114]. Studies show that KP infection suppresses PS exposure on apoptotic neutrophils, preventing effective recognition and phagocytosis by macrophages, thereby increasing bacterial spread [118]. Therefore, enhancing apoptotic signaling or inhibiting KP capsule-mediated immune evasion could improve macrophage bactericidal efficacy against KP.

#### Pyroptosis

Pyroptosis is a newly discovered form of programmed cell death, initially termed as caspase-dependent inflammatory cell death and later redefined as gasdermin-mediated programmed cell death, with the gasdermin family being the executioners of pyroptosis [119, 120]. In 2015, Shao Feng et al. found that caspase-1/11/4/5 can induce pyroptosis by cleaving a protein called GSDMD to release its N-terminal domain, thereby initiating pyroptosis [121]. Pyroptosis is mainly characterized by continuous cell swelling until the cell membrane ruptures, leading to the release of cellular contents and activation of robust inflammatory responses, mediated by both the classical caspase-1–GSDMD and nonclassical caspase-11–GSDMD pathways [119–121]. In the classical pathway, caspase-1 activation is driven by inflammasomes such as NLRP3, Pyrin, NLRP1b, NLRC4, and AIM2 [122]. It is noteworthy that, while pyroptosis is part of the host defense mechanism, if infected pyroptotic cells are not promptly cleared by the host, they may promote pathogen dissemination, and excessive pyroptosis may exacerbate tissue damage [123].

KP can induce pyroptosis in macrophages through the classical pathway, activating NLRP3-caspase-1 and leading to the cleavage and release of downstream IL-1β [34, 44, 47]. The specific mechanism involves the ability of KP’s CPS and LPS to induce mitochondrial membrane permeability changes and mitochondrial ROS generation, mediating the activation of the NLRP3 inflammasome [34]. It is noteworthy that K1-CPS-induced NLRP3 activation is associated with increased ROS generation via the TLR4 pathway, enhanced mitogen-activated protein kinase phosphorylation, and NF-κB activation [34, 44]. In addition to activating the NLRP3 inflammasome, KP can also induce caspase-1-mediated pyroptosis via Pyrin inflammasome activation [124]. KP infection upregulates CDC42-165aa in alveolar macrophages (primary and MH-S cells), promoting Pyrin inflammasome hyperactivation and severe lung injury in intranasally infected mice [124]. Additionally, considering that intracellular LPS can activate caspase-11, leading to nonclassical pyroptosis, and since KP can enter macrophages, it suggests that caspase-11 may also be involved in the process of KP-induced pyroptosis [125–127]. Evidence shows that caspase-11-deficient BMDM cells successfully block KP induced IL-1β secretion and pyroptosis, indicating that KP can activate caspase-11-mediated nonclassical pyroptosis [125–127]. Besides NLRP3, NLRC4 is a member of the NOD-like receptor family, which can also undergo self-oligomerization and recruit caspase-1 to form inflammasomes, triggering cell pyroptosis [34, 45, 50]. Studies have found that KP can activate the NLRC4 inflammasome through its capsular polysaccharide (CPS) and LPS, which is crucial for inducing IL-1β generation [34, 45]. Interestingly, recent studies have reported on MDR-KP resistant to imipenem, which, after inappropriate use of imipenem, enhances the expression of GroEL in bacterial OMVs, promoting their interaction with the receptor LOX-1, thereby initiating downstream macrophage pyroptosis pathways and the release of the pro-inflammatory cytokine IL-1β [58].

The role of cell pyroptosis in KP infection is complex. Studies have shown that mice lacking the NLRP3 gene exhibit higher mortality and lower lung inflammation after KP infection [34, 44, 47]. Additionally, Caspase-11(−/−) mice exhibit impaired neutrophil recruitment and bacterial clearance in the early stages of KP infection, increased sensitivity to KP infection, and pretreatment with IL-1α neutralizing antibodies inhibits neutrophil recruitment and bacterial clearance in wild-type mice [125, 126]. Caspase-11-deficient mice show significantly reduced fibrin formation in the lungs following KP infection, disrupting mouse coagulation function, and increased bacterial counts in the lungs, although no changes in invasive dissemination of KP and organ dysfunction [125, 126]. Furthermore, NLRC4 and IL-1R1-deficient mice exhibit lower survival rates and decreased expression of cytokines/chemokines in the lungs following KP infection, while exogenous IL-1β partially rescues the high mortality of NLRC4-deficient mice [34, 45]. However, in addition to this, studies have also found that KP-mediated cell pyroptosis can exacerbate tissue damage and mortality. In the case of MDR-KP infection, inappropriate antibiotic use exacerbates mortality in infected mice by promoting excessive cell pyroptosis [58]. Additionally, PSPAs, naringin and chlorogenic acid can alleviate lung damage, limit KP dissemination, and improve survival rates by inhibiting NLRP3 inflammasome activation [47, 128, 129]. Moreover, *circCDC42* KO enhances survival in KP-infected mice following intranasal inoculation by suppressing Pyrin inflammasome activation, reducing bacterial load in the bloodstream, decreasing immune cell infiltration in the lungs, and alleviating lung injury [124]. In summary, the role of cell pyroptosis in KP infection remains unclear and requires further clarification, possibly through KO mice of the gasdermin family as pyroptosis executioner.

#### Necroptosis

Necroptosis is a programmed form of necrosis that occurs when cells fail to undergo apoptosis [8]. Specifically, during the initiation of apoptotic signaling, if caspase-8 activity is inhibited after the binding of TNF-α to TNFR1 on the macrophage surface, it leads to the suppression of RIPK1 cleavage [8, 130]. This suppression allows a series of auto- and trans-phosphorylation events between RIPK1 and RIPK3, resulting in the recruitment and subsequent phosphorylation of MLKL [8, 131, 132]. Phosphorylated MLKL translocates to the plasma membrane, where it forms pore complexes, causing membrane rupture and cell death.

KP has been shown to activate the necroptosis pathway [117, 133, 134]. In a 2017 study by Danielle Ahn et al., it was observed that Ripk3−/− mice exhibited reduced bacterial loads in bronchoalveolar lavage fluid and lungs, along with decreased levels of the inflammatory cytokine TNF-α [134]. Additionally, in mice lacking RIPK1 kinase activity, bacterial clearance did not significantly differ from WT mice when infected with KP [134]. This suggests that RIPK3 might play a more critical role in the necroptosis pathway during KP infection, although the exact mechanisms remain unclear. Further studies have reported that KP can inhibit the activation of apoptosis and instead trigger necroptosis in neutrophils, thereby evading macrophage-mediated efferocytosis of KP-infected neutrophils [114]. Pharmacological inhibition of RIPK1 and RIPK3 has been shown to restore the efferocytic uptake of infected neutrophils by macrophages [114].

In conclusion, KP-induced necroptosis contributes to its pathogenicity by evading immune clearance mechanisms. Inhibiting necroptosis presents a promising approach to bolster the host immune response against KP, potentially improving bacterial clearance and reducing infection severity [114, 134]. Further research into the specific roles and regulatory mechanisms of RIPK3 in necroptosis could lead to more effective treatments for KP infections.

### Perspectives in treatment

While appropriate antimicrobial agents are crucial for eradicating KP and mitigating the release of PAMPs, immunotherapeutic strategies targeting macrophage responses post-KP infection offer a complementary approach. These immunomodulatory interventions have the potential to attenuate the ongoing inflammatory cascade triggered by DAMPs, thereby mitigating tissue damage. This review provides a comprehensive analysis of emerging immunotherapeutic modalities that address the complex interplay between antimicrobial efficacy and host immune response in KP infections. By simultaneously addressing pathogen elimination and host immune regulation, this dual-pronged approach may enhance therapeutic outcomes in severe KP infections. This section provides an overview of promising therapeutic interventions, aimed at improving clinical outcomes for patients with KP infections.

### Using antibodies/vaccines to prevent KP from evading macrophage immunity

The improper use of antibiotics can eliminate beneficial gut bacteria, leading to dysbiosis, emphasizing the need for targeted antibiotic therapies to avoid broad-spectrum antibiotics [135]. Designing antibodies or vaccines based on specific epitopes of KP might achieve targeted treatment without harming beneficial gut bacteria [136, 137]. Extensive experimental evidence indicates that the use of specific antibodies or vaccines significantly enhances macrophage uptake and killing of KP, thereby improving recovery and survival rates in infection [136, 138–140]. Currently, bacterial vaccines containing inactivated KP are undergoing phase 2 clinical trials for the prevention of recurrent urinary tract infections, with 78% of patients experiencing no subsequent infections within a year, among those who had three or more infections in the past year [141]. Vaccine design for KP primarily focuses on CPS, LPS, and OMPs.

#### CPS

Specific IgG antibodies against the K1 serotype CPS of KP have been designed, finding that prophylactic use increases macrophage uptake of KP and inhibits sepsis and lung infections in mice [73, 142]. Elizabeth Diago-Navarro et al. identified monoclonal antibodies 17H12 and 8F12 targeting various CPS types, including K1, K2, K3, and K16, which effectively promote macrophage opsonophagocytosis and intracellular killing, reducing bacterial dissemination to organs [138]. Even in nonhuman primate models such as crab-eating macaques, CPS-based vaccines significantly reduce the severity of pneumonia caused by CRKP [143].

#### LPS

The O-antigen region of LPS is the most variable part, categorizing KP into nine subtypes. Studies have found that specific monoclonal antibodies against O1 or O2 serotypes reduce mortality in acute pneumonia mouse models by enhancing phagocytic killing [144]. Hegerle et al. developed a novel vaccine comprising the four most common O-antigens (O1, O2, O3, and O5) of KP, demonstrating its protective effect against infection and reducing bacterial load [136]. Another study evaluated the protective effect of monoclonal antibody A1102 against the LPS O antigen of a MDR-KP ST258 strain, finding that it enhances phagocytic killing [143]. Recently, Rehab Bahy et al. used a modified hot phenol method to extract LPS from clinical isolates of KP and prepared a vaccine, discovering its significant protective effect [145].

#### OMPs

The use of vaccines targeting LPS and CPS presents limitations due to the structural variability among different strains of KP. Although vaccines covering multiple O or K serotypes have been developed, their coverage remains restricted [136, 138]. Considering the conserved and crucial nature of outer membrane proteins OmpA and OmpK36 in KP, Litty Babu et al. designed a recombinant multivalent vaccine consisting of OmpA and OmpK36 domains (r-AK36 protein). This vaccine demonstrated that AK36 effectively protects mice from death due to KP infection, and pre-infection administration of their antibodies also showed significant protective effects [139]. Considering that targeting OMPs can provide protection against lethal doses in mice, developing corresponding vaccines holds promising potential.

#### OMVs

OMVs derived from KP also provide mice with protection against lethal KP infection [146]. Studies have shown that OMV vaccination offers complete protection in a dose-dependent manner against severe or fatal disease in mouse bacteremia models [146]. However, considering the complexity of OMV components, further investigation is required. As previously mentioned, the GroEL protein in OMVs of KP can interact with the receptor LOX-1, promoting the release of large amounts of LPS into cells and leading to severe pyroptosis-mediated tissue damage [58]. Therefore, a deeper understanding of OMVs is essential for developing safe and effective vaccines.

### Enhancing macrophage phagocytic killing in early infection

Considering that KP can persist and replicate within macrophages, interventions that enhance early macrophage killing of KP may offer new therapeutic avenues [16, 18, 94]. A recent report indicated that STAT6 is crucial for macrophage activation, and STAT6 deficiency aids macrophage clearance of KP [17]. Inhibiting STAT6 may therefore be a promising target in KP treatment [17]. Additionally, enhancing autophagy represents another potential immunotherapy. Inhibition of autophagy with bafilomycin A1 reduces bacterial clearance increases lung injury, and lowers survival rates [104, 110]. Conversely, treatment with Beclin-1-activating peptide enhances macrophage autophagy, significantly mitigating adverse outcomes of KP-induced sepsis [111]. Also, 1,25(OH)_2_D_3_ can induce autophagy to enhance macrophages in clearing KP [110]. The role of pyroptosis in KP infection is complex; deficiency in NLPR3, caspase-11, NLRC4, or IL-1R1 results in higher mortality, suggesting that early pyroptosis enhances macrophage phagocytic killing [44, 125, 127]. Early application of pyroptosis activators may thus have therapeutic potential.

### Inhibiting pyroptosis and necroptosis in late infection to prevent inflammation and bacterial spread

The pro-inflammatory nature of macrophage pyroptosis and necroptosis aids in fighting bacteria, but excessive cell death can release intracellular components and cytokines, causing severe inflammation and tissue damage [6, 8, 120]. Recent studies show that inappropriate antibiotic use against MDR-KP increases lung tissue inflammation and damage by promoting macrophage pyroptosis while inhibiting pyroptosis lowers mortality [58]. Additionally, PSPAs, naringin, and CGA can inhibit NLRP3 inflammasome-mediated pyroptosis, limiting KP infection spread and improving survival rates [47, 128, 129]. For necroptosis, Ripk3−/− mice exhibit lower bacterial loads in bronchoalveolar lavage fluid and lungs, reduced TNF-α levels, and less tissue damage [134]. Therefore, inhibiting pyroptosis and necroptosis during late infection could be a promising therapeutic strategy. A recent study found that the nonsteroidal anti-inflammatory drug acetylsalicylic acid (ASA), an inhibitor of the cyclooxygenase-1 enzyme, effectively reduces mortality caused by the clinical hvKP strain 17ZR101, confirming the critical role of cytokine storms in hvKP infection-related deaths [147]. Furthermore, considering that preventing cytokine storm-induced septic shock does not eradicate KP from the host, the study investigated the combined therapy of 5-aminosalicylic acid (5-ASA) and the antibiotic ceftazidime–avibactam [147]. The results demonstrated that this combination therapy reduces inflammation mediated by infiltrating immune cells and decreases bacterial load [147].

## Conclusions

We explored the mechanisms of KP recognition through various PRRs, and macrophages utilize these receptors to activate a cascade of intracellular pathways. Additionally, we examined the various cell death pathways such as autophagy, apoptosis, pyroptosis, and necroptosis. Notably, recent studies revealed KP’s dual extracellular/intracellular existence, forming M(Kp). This intracellular persistence is facilitated by the accumulation of anti-inflammatory metabolites such as itaconate and cytokines such as IL-10, which prevent effective clearance of KP.

Considering the limited clinical treatment options, it is crucial to understand the macrophage response to KP. Autophagy and apoptosis are generally beneficial for bacterial clearance. The fusion of KP-containing phagosomes with lysosomes facilitates bacterial killing, while apoptosis effectively recruits immune cells such as neutrophils to phagocytose and kill KP.

However, excessive inflammation can have detrimental effects, especially if pyroptosis and necroptosis occur, leading to the production of large amounts of inflammatory cytokines. These mediators can cause inflammatory damage to surrounding tissues, releasing substantial DAMPs, which further amplify inflammation and lead to multiple organ failure. Therefore, in cases of sepsis or septic shock induced by KP infection, inhibiting the key mediators of pyroptosis and necroptosis pathways to prevent the inflammatory storm could be beneficial. Interestingly, using antibiotics to kill bacteria and inhibit their growth, thus blocking the release of PAMPs, combined with anti-inflammatory agents to prevent tissue damage and DAMPs release, could be an effective approach. Research has demonstrated that ASA, an anti-inflammatory drug, combined with meropenem, can protect mice from lethal doses of KP.

Based on these insights, our analysis underscores the potential for an integrative therapeutic approach that combines antimicrobial therapy, immunomodulation, and anti-inflammatory interventions. This strategy aims to simultaneously target pathogen elimination, host immune regulation, and tissue protection. Specifically, we propose utilizing targeted antibiotics to eradicate KP and suppress PAMPs release, modulating macrophage responses through enhancement of autophagy and apoptosis for rapid KP clearance, and selectively inhibiting pyroptosis and necroptosis in severe infections to prevent inflammatory cascades. Additionally, the combination of antibiotics with anti-inflammatory agents shows promise in attenuating tissue damage and limiting DAMPs release. This integrative approach offers a promising avenue for improving treatment outcomes in KP infections, especially those involving MDR and hypervirulent strains, potentially revolutionizing our management strategies for these challenging infections.

## Data Availability

Not applicable.

## Abbreviations

CPS: Capsular polysaccharide
KP: Klebsiella pneumoniae
PAMPs: Pathogen-associated molecular patterns
PRRs: Pattern recognition receptors
DAMPs: Damage-associated molecular patterns
CRKP: Carbapenem-resistant KP
LPS: Lipopolysaccharides
OMPs: Outer membrane proteins
OMVs: Outer membrane vesicles
TLRs: Toll-like receptors
NLRs: Nucleotide-binding oligomerization domain-like receptors
RLRs: Retinoic acid-inducible gene I-like receptors
CLRs: C-type lectin receptors
ALRs: AIM2-like receptor
cGAS: Cyclic GMP–AMP synthase
LppA: Murein lipoprotein
APG: Acylpolygalactosyl
PSPAs: Purple sweet potato anthocyanins
T3SS: Type III secretion system
T6SS: Type VI secretion system
MMR: Macrophage mannose receptor
LOX-1: Lectin-like oxidized low-density lipoprotein receptor-1
STING: Stimulator of interferon genes
TBK1: TANK-binding kinase 1
IRF3: Interferon regulatory factor 3
IFNAR: Interferon-alpha/beta receptor
ATGs: Autophagy-related genes
MDR: Multidrug resistant
